# Case report: Misdiagnosis of accessory spleen in the left adrenal region as an adrenal tumour after splenectomy

**DOI:** 10.3389/fsurg.2022.1017603

**Published:** 2022-10-17

**Authors:** Yuhua Zou, Xiaojuan Xie, Sheng Yan, Gengqing Wu, Quanliang Liu

**Affiliations:** ^1^ Department of Urology, The First Affiliated Hospital of Gannan Medical University, Ganzhou, China; ^2^ Department of Cardiology, The First Affiliated Hospital of Gannan Medical University, Ganzhou, China

**Keywords:** accessory spleen, adrenal tumour, splenectomy, misdiagnosis, image diagnosis, case report

## Abstract

**Background:**

Adrenal tumours are common in urology and endocrinology, and the diagnosis of adrenal tumours were mainly depends on imaging diagnosis. Howerver, misdiagnosis can still occur for some adrenal space-occupying lesions without specific manifestations or abnormal biochemical indexes.

**Methods:**

We report the case of a 55-year-old patient with a soft-tissue mass in the left adrenal region, and have no specific manifestations or abnormalities in biochemical indexes. The patient had undergone open splenectomy 20 years ago for splenic rupture caused by traffic-accident trauma, and had a 10-year special history of hypertension. Because of the uncertain nature of the mass, surgical treatment was recommended.

**Results:**

The surgeon managed to remove the left adrenal region mass. During the surgery, the adrenal source was excluded. In the histological examination, the splenic corpuscle and splenic medullary structure were seen under the microscope, and an accessory spleen was diagnosed.

**Conclusions:**

The accessory spleen was located in the adrenal region rarely, and can easily be misdiagnosed as an adrenal tumour. When the cases show abnormal adrenal space-occupying lesions in imaging examinations, non-adrenal diseases should be considered. we need to combine different imaging techniques for analysis, and think more about it, avoid misdiagnosis leading to unnecessary surgery.

## Introduction

Adrenal tumours are common in urology and endocrinology (1–3). With the continuing development of different imaging techniques, such as ultrasound, computed tomography (CT) scan, magnetic resonance imaging (MRI), and detection techniques for adrenal hormone, the accuracy in localisation and qualitative diagnosis of adrenal tumours has gradually increased. However, misdiagnosis can still occur for some adrenal space-occupying lesions without specific manifestations or abnormal biochemical indexes. Clinical staff need a comprehensive analysis method to reduce unnecessary surgery. We report a case of patient who was misdiagnosed of accessory spleen in the left adrenal region as an adrenal tumour after splenectomy.

## Case presentation

The reporting of this study conforms to CARE guidelines (4, 5). In Dec. 6, 2021, a 55-year-old patient was admitted to the department of emergency due to fallen down while working, resulting in thoracic and abdominal pain and discomfort. Subsequently, he visited the emergency department of emergency for an abdominal CT scan, which showed a left-adrenal space-occupying lesion. Consequently, enhanced scanning and hospitalisation were recommended. The patient had undergone open splenectomy 20 years ago for splenic rupture caused by traffic-accident trauma. The patient had a 10-year special history of hypertension, and his blood pressure was well controlled by regular oral antihypertensive drugs.

**Figure 1:**
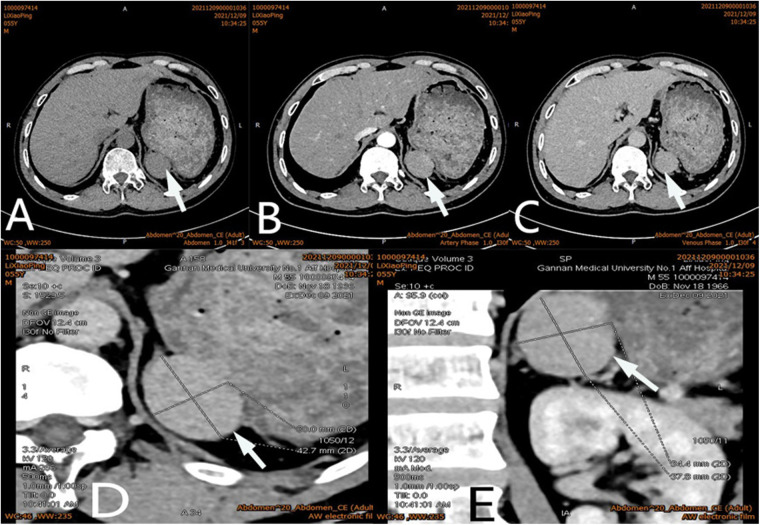
Ct scan of a mass (arrow) in the left adrenal region. The CT value of plain scanning which the internal density was heterogeneity (**A**), and enhanced scanning presented moderate enhancement (**B**), the delayed phase of the mass (**C**). Coronal image of the mass (**D**). Sagittal image of the mass (**E**).

**Figure 2:**
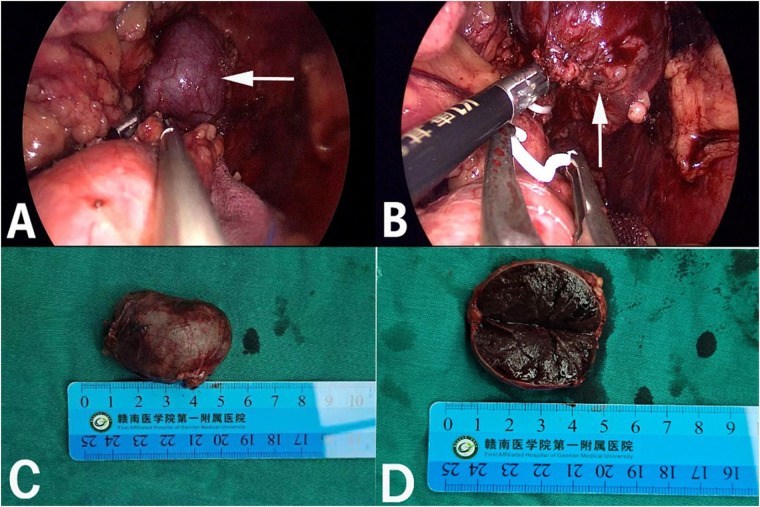
Retroperitoneal laparoscopic resection of the mass (arrow). The mass is located in the left suprarenal region, and the boundaries between the dorsal side of the mass were clear, loose and non-adhered (**A**), the ventral side adhered closely to the peritoneum (**B**). Gross appearance. The mass was dark red, and the surface envelope of the mass was intact (**C**), and had a similar splenic structure with multiple sinusoid tissues (**D**).

**Figure 3:**
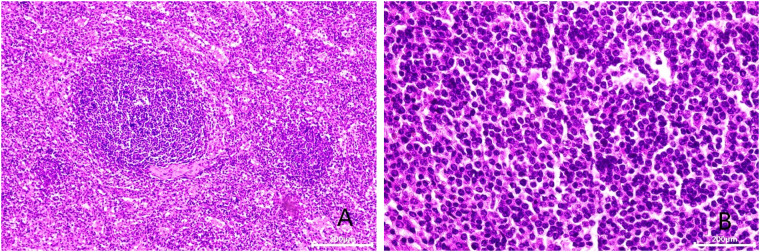
Microscopic appearance (**A,B**). Histologic magnification revealed accessory splenic tissue [hematoxilyn and eosin staining, ×200 (**A**), ×400 (**B**)].

After admission, his maximum monitored blood pressure was 138/87 mmHg (1 mmHg = 0.133 kPa), and his monitored heart rate ranged from 86 to 103 bpm. The patient denied a history of diabetes, centripetal obesity or specific manifestations of cortisol signs, such as full-moon face and buffalo hump. All biochemical tests, including blood potassium: 3.57 mmol/L; cortisol (0AM): 1.67 µg/dl; cortisol (8AM): 7.79 µg/dl; cortisol (16PM): 4.46 µg/dl; adrenocorticotropic hormone (0AM): 2.47 pmol/L; adrenocorticotropic hormone (8AM): 3.76 pmol/L; adrenocorticotropic hormone (16PM): 2.97 pmol/L; renin (standing position): 41.68 pg/ml; aldosterone (standing position): 141 pg/ml; renin (recumbent position): 16.07 pg/ml; aldosterone (recumbent position): 129 pg/ml, and 24-h urinary 3-methoxy-4-hydroxymandelic acid, 24-h urinary catecholamines were normol. Ultrasound (US) was performed which identified a 42 × 30 × 35 mm adrenal mass in the left-adrenal region. In order to confirm the diagnosis, computed tomography (CT) scan was conducted, the CT scan confirmed the US findings, showing a 42 × 28 × 36 mm soft-tissue mass shadow in the left adrenal region, and the internal density was heterogeneity (Figures 1A,D,E). The CT value of plain scanning was 36 to 68 HU, and enhanced scanning presented moderate enhancement (Figure 1B). The CT value in the arterial phase was 62 to 104 HU, and that in the delayed phase was 46–89 HU (Figure 1C). The lesion was considered to be a benign neoplastic lesion in the left adrenal region, possibly a left-adrenal adenoma or pheochromocytoma. According to the 2019 CUA Guidelines for Urinary Surgery (6), suspected non-functioning adrenal adenoma, which is defined as an adrenal tumour ≥3 cm, surgical treatment was recommended. The patient was placed in the right lateral decubitus position and an retroperitoneal laparoscopic approach was performed. Intraoperatively, a round dark-grey mass was observed on the upper ventral region of the retroperitoneal left kidney, with a size of about 40 mm and a surface completely covered with smooth membranous peritoneum-like tissue. The boundaries between the dorsal side of the mass and upper pole of the left kidney, left adrenal gland and surrounding adipose tissue were clear, loose and non-adhered (Figure 2A). The ventral side adhered closely to the peritoneum and provided blood supply. The mass was completely resected after partial removal of the adhered peritoneum and blocking of blood vessels (Figure 2B). The vital signs of the patient showed no significant fluctuations during tumour separation, compression and resection. The anatomopathological exam revealed a 40 × 30 mm in size, which the surface envelope was intact, and the mass was dark red and had a similar splenic structure with multiple sinusoid tissues (Figures 2C,D). In the histological examination, the splenic corpuscle and splenic medullary structure were seen under the microscope, and an accessory spleen was diagnosed (Figures 3A,B). No perioperative complications were registered and remained in the hospital for three days after surgery. The patient was satisfied with the treatment. Postoperatively, the patient was followed up for 3 months, and no lumbago or infection was observed.

## Discussion

Adrenal tumours are common in urology and endocrinology (1–3). The common clinical manifestations are secondary to excessive secretion of adrenal-gland-related hormones, such as hypertension, hyperglycaemia, myasthenia, puffiness, full-moon face, buffalo hump and irregular menstruation, but some patients may have no specific clinical manifestations (7). Clinical localisation and diagnosis of an adrenal tumour are mainly based on imaging examinations, such as ultrasound, CT and MRI. Because of the deep and hidden adrenal region, like any imaging method, an ultrasound examination is likely to be disturbed and entail the risk of misdiagnose. It is the most subjective and require considerable clinical experience and knowledge of sonographers for proper interpretation (8). Adrenal tumours often show uniform low-density shadows on CT scan, whereas an accessory spleen usually presents with uniform density, and its enhancement effect in the arterial and venous phases is always similar to that of the spleen. Our patient had undergone splenectomy 20 years ago, so we could not compare it with the normal spleen. In addition, the CT morphology of the accessory spleen in this case was similar to that of a benign adrenal tumour, with a regular shape, a clear boundary and a capsule. Moreover, The CT value of plain scanning was 36 to 68 HU, and enhanced scanning presented moderate enhancement (Figure 1B). The CT value in the arterial phase was 62–104 HU, and that in the delayed phase was 46–89 HU (Figure 1C). The CT findings were similar to those for an adrenal mass. Those findings, combined with the patient's history of hypertension, led to our suspicion of a left-adrenal tumour. Thus, surgical exploration was unavoidable.

Accessory spleen, also called supernumerary spleen, refers to tissue with the same structure and function as the normal spleen that exists in addition to the normal spleen, and it is a common congenital anatomical abnormality, with an incidence of 10%–30% (9). Accessory spleen formation is usually caused by failed fusion of some parts of an embryonic spleen bud in the mesogastrium or by independent development of partial spleen tissue that detaches from the spleen (9, 10). The location, number and size of the accessory spleen can also vary. A single accessory spleen is common, but multiple accessory spleens may also occur, and generally its diameter is 10–40 mm (11). Accessory spleen often occurs in the splenic hilum, but also in other parts of the abdomen, such as the pancreatic tail, greater omentum, hepatogastric ligament, splenogastric ligament space, gastric wall, intestinal wall and even pelvic reproductive organs, and the retroperitoneal position is extremely uncommon (12, 13). When accessory spleen was located in the adrenal region, the accessory spleen can easily be misdiagnosed as an adrenal tumour (14, 15). Compared with the left-retroperitoneal accessory spleen, the right-retroperitoneal accessory spleen is rarer but more likely to be misdiagnosed as an adrenal tumour (15).

At present, it is generally recognised that Tc-99m-labelled heat-denatured red-blood-cells scintigraphy is a reliable, sensitive and non invasive imaging method for confirm the qualitative clinical diagnosis of accessory spleen (16, 17). The diagnostic principle is that the reticuloendothelial cells of the spleen can selectively absorb and destroy the function of damaged and denatured red blood cells (RBC). Firstly, *in vitro* ^99m^Tc-labelled heat-denatured red blood cells (^99m^Tc-DRBC) are intravenously injected into the human body and then absorbed by the spleen, followed by the liver and reticuloendothelial tissues, such as bone marrow. In these tissues, haem in RBC is destroyed, digested and metabolised to form bilirubin, so only the spleen can aggregate ^99m^Tc-DRBC, and has a high-uptake rate, followed by the liver and bone marrow, but other tumour tissues and lymph glands cannot. The radioactive concentration per unit volume is 2–3 times higher in the spleen than in the liver. False-negative results only are obtained in accessory spleens which were the relatively small sizes because there is no accumulation of radioactive tracers (18). ^99m^Tc-DRBC scintigraphy can well distinguish ectopic accessory spleen from tumour recurrence, new tumours and enlarged lymph nodes, with high accuracy (19). It is a useful nuclear medicine method to solve some of the clinical puzzles, but its development is limited in China. Moreover, the main blood supply of the accessory spleen in the adrenal region is from splenic artery branches. Careful distinguishing between accessory spleen and adrenal tumours from the perspective of blood supply in imaging may reduce misdiagnosis to a greater extent. Therefore, when encountering suspected cases in clinics, we need to combine different imaging techniques for analysis, and think more about it, avoid misdiagnosis leading to unnecessary surgery.

Generally, the accessory spleen has no clinical symptoms and is mostly detected by physical examination or other examinations (20). Currently, it is believed that the accessory spleen needs no special treatment and requires resection only for rupture, infarction or vascular torsion that causes corresponding clinical symptoms (21). Additionally, the normal spleen and accessory spleen need to be resected together in the treatment of haematological diseases, such as idiopathic thrombocytopenic purpura, otherwise may cause recurrent disease (22). However, an enlarged accessory spleen is often clinically misdiagnosed as a tumour or enlarged lymph node for surgical treatment (23). Our patient had undergone open splenectomy for splenic rupture caused by a traffic-accident trauma 20 years ago. Due to the long time and no corresponding imaging data as a preoperative reference, we could not determine that the accessory spleen of this patient was caused by compensatory hypertrophy of the accessory spleen after splenectomy or its ectopic implantation during splenectomy. Intraoperatively, the boundaries between the dorsal side of the mass and the kidney, adrenal gland and surrounding adipose tissue were clear, loose and non-adhered (Figures 2A), but the ventral side adhered closely to the peritoneum (Figures 2B). It can be seen that the lateral peritoneum was not opened during splenectomy. Therefore, in this case, the accessory spleen was mostly caused by compensatory hypertrophy of the accessory spleen after splenectomy. After resection of the normal spleen, the ectopic accessory spleen can develop compensatory hypertrophy and play the role of the normal spleen. After splenectomy, resulting in decreased immune cells, weakened and imbalanced regulation of the immune system and increased infection, which would require special attention.

In this case, because of our lack of experience, the patient underwent unnecessary surgery. We analysed the causes of our misdiagnosis and had to admit that different imaging techniques for the suspected case were not performed, and the single examination had some defects. Additionally, this patient had a history of splenectomy, and the accessory spleen was located in the adrenal region, which is relatively rare. The patient also had a history of hypertension, which greatly interfered with the preoperative diagnosis, but the main reason was the lack of understanding of accessory spleen before surgery. During the surgery, the adrenal source was excluded, we also questioned the source of the tumour. To ensure the integrity of the tumour and the principle of “no tumour”, we did not perform a frozen-section examination. However, it would be worth discussing whether a better outcome can be obtained if such tumours are subjected to frozen-section examination during surgery.

## Conclusions

In summary, the diagnosis of space-occupying lesions in the left-adrenal region remains challenging, especially in some patients who have no specific manifestations or abnormalities in biochemical indexes. When the cases show abnormal adrenal space-occupying lesions in imaging examinations, non-adrenal diseases should be considered. we need to combine different imaging techniques for analysis, and think more about it, avoid misdiagnosis leading to unnecessary surgery.

## Data Availability

The raw data supporting the conclusions of this article will be made available by the authors, without undue reservation.
